# Increasing evidence supporting fecal calprotectin for distinguishing Crohn's disease perianal fistulas from cryptoglandular fistulas

**DOI:** 10.1002/ueg2.12646

**Published:** 2024-11-07

**Authors:** Samuel Raimundo Fernandes, Inês Coelho Rodrigues

**Affiliations:** ^1^ Serviço de Gastrenterologia e Hepatologia Unidade Local de Saúde de Santa Maria EPE Lisboa Portugal; ^2^ Clínica Universitária de Gastrenterologia da Faculdade de Medicina de Lisboa Lisboa Portugal; ^3^ Grupo de Estudos de Doença Inflamatória do Intestino GEDII Porto Portugal

**Keywords:** Crohn's disease, fecal calprotectin, perianal fistulas

Perianal fistulizing disease remains a challenging condition complicating up to one third of patients with Crohn's disease (CD).^1^ Perianal CD is a predictor of disabling disease significantly impairing the psychological, physical and sexual life of patients. It also associates with an increased risk of immunosuppression, hospitalization and surgery.^2^, ^3^, ^4^ One of the challenges in managing patients with perianal disease is distinguishing cryptoglandular from CD fistulas. Most patients with perianal CD will present concomitant intestinal inflammation. Nevertheless, roughly 10%–15% of patients will not display signs of endoscopic activity at presentation.^4^, ^5^ Cryptoglandular fistulas are primarily managed surgically. In CD, however, there is extensive evidence supporting a combination of surgical and medical treatment, especially using tumor necrosis factor antagonists.^3^, ^6^ Given the significantly different management strategies for these two fistula types, an accurate diagnosis is of the utmost importance. Inadequate surgical procedures may result in significant morbidity including a risk of fecal incontinence, defunctioning stoma and protectomy.^7^ In the current study, Becker and colleagues investigate the role of fecal calprotectin and calprotectin scrappings from the fistula tract in distinguishing CD fistulas from cryptoglandular fistulas. The authors found higher levels of fecal calprotectin in patients with CD compared to cryptoglandular fistulas (354.3 μg/g vs. 47.3 μg/g; *P* = 0.003) with a non‐significant trend in patients without luminal disease (93 μg/g vs. 47.3 μg/g, *P* = 0.058). The resulting model was fairly accurate in distinguishing these two types of fistulas with an area under the ROC curve of 0.733 (95% CI [0.644–0.903]). A cut‐off of fecal calprotectin of 344 μg/g differentiated CD from cryptoglandular fistulas with a positive predictive value of 96% (albeit only a negative predictive value of 39%). On the other hand, the authors did not find a role for calprotectin in scrappings of the fistula tract in distinguishing these two conditions. Nevertheless, the biomarker was able to predict prognosis using the TOpCLASS classification, essentially identifying patients more amenable for surgical repair.

These results are in line with another retrospective study including 56 patients (37 with CD and 19 with cryptoglandular fistulas).^8^ The authors found higher levels of fecal calprotectin in patients with CD (708 μg/g vs. 32 μg/g; *P* < 0.001), even after restricting analysis to patients in endoscopic remission (238 μg/g vs. 32 μg/g; *P* < 0.001). However, their cutoff was significantly lower (>150 μg/g) and with higher positive and negative predictive value (94% and 71%, respectively). The results of the current study may be explained by an underestimation of disease activity in the rectum due to the use of bowel ultrasound in patients without an ileocolonoscopy.

While the study adds to our understanding of this often misrepresented condition, the accurate differentiation between these two conditions remains a challenge. In a recent systematic review magnetic resonance imaging detection of rectal inflammation, multiple‐branched fistula tracts, and abscesses presented high specificity (92%, 95%, and 81%) but low sensitivity (37%, 17%, and 27%) in separating CD fistulas from cryptoglandular fistulas.^9^ Similar results were found using endoanal ultrasound. The review also found limited evidence using capsule endoscopy to detect small bowel lesions, cytokine, immune cell, microbiome, and metabolic profiles in the fistula and rectal biopsies. Given that none of these modalities appear sufficiently sensitive to be relied on as sole diagnostic tests, it is likely that a combination of different methods will be more helpful in patients with a negative ileocolonoscopy. At the present date, fecal calprotectin, capsule endoscopy, and pelvic imaging appear to be our best options in differentiating these conditions.

## CONFLICT OF INTEREST STATEMENT

The authors have no conflicts of interest or funding to declare related to the present study.

## Data Availability

Data is available upon request.
